# Successful treatment of refractory hughes stovin syndrome with infliximab

**DOI:** 10.1186/1546-0096-12-S1-P352

**Published:** 2014-09-17

**Authors:** Giulia Gortani, Meta Starc, Martina Tubaro

**Affiliations:** 1 Pediatrics, IRCCS Burlo Garofolo, Trieste, Italy

## Introduction

Hughes-Stovin Syndrome (HSS) is a very rare clinical disorder characterized by pulmonary artery aneurysms and peripheral venous thrombosis. Typical symptoms are recurrent fever, chills, haemoptysis and it usually affects young men. The natural course of the illness is usually fatal because of fulminant haemoptysis. The aetiology of Hughes-Stovin syndrome is still unknown; however it is supposed to be a clinical variant manifestation of Behçet disease.

## Objectives

While several case reports have shown infliximab to be efficacious in patients with Behçet's syndrome and pulmonary arterial aneurysm, no experience exists about it’s use in Hughes Stovin Syndrome.

## Methods

In the present report we describe the first successful use of infliximab in refractory Hughes Stovin Syndrome.

## Results

A 12-year-old albanian boy was first admitted to local a Children Hospital with fever, diplopia, headache and repeated vomiting lasting for weeks. There was no prior medico-surgical illness history. Cerebral MRI (magnetic resonance imaging) revealed thrombosis of right sigmoid and transverse sinuses. Acetyl salicylic acid treatment was started and the boy was discharged. Shortly therafter he started complaining high fever and left leg pain. Computed tomography (CT) angiography revealed a right femoral artery pseudoaneurysms. Echocardiography showed a large tricuspid valve vegetation. In the suspicion of bacterial endocarditis antibiotic therapy was instituted, without improvement of symptoms. Because of refractory fever the boy was so referred to our Children Hospital. Inflammatory markers were markedly elevated. Echocardiography confirmed the presence of a tricuspid valve vegetation and detected a large atrial thrombus. The boy was put in heparin and referred to a Cardiothoracic Surgery Department, where cardiac thrombus and vegetations were surgically removed. Nevertheless, fever persisted. Thoracic angio-CT-scan was so performed, that showed three partially thrombosed aneurysms of right segmental pulmonary arteries. On the basis of pulmonary artery aneurysms, sinus thromboses and femoral pseudoaneurysm a diagnosis of Hughes-Stovin syndrome was made. HLA B-51 allele was detected. There where no other findings consistent with Behçet disease. Pulse intravenous cyclophosphamide plus oral prednisone was started with prompt amelioration of symptoms and disappearance of fever. Antiaggregation and coagulation were discontinued to avoid the risk of hemoptysis. Two months thereafter pulmonary aneurysms resulted only partially reduced and inflammatory markers were still slightly elevated. Episodes of relapsing remitting fever occurred. His treatment was so changed to intravenous infliximab with methotrexate to which he showed an excellent response with complete regression of aneurysms at contrast enhanced pulmonary MRA and normalization of inflammatory markers. This permitted successful and quick steroid tapering.

## Conclusion

Anti-TNF inhibitors should be considered in patients affected by Huges Stovin Syndrome who do not respond to treatment with corticosteroids and cyclophosphamide

## Disclosure of interest

None declared

